# Efficient Elimination of Viruses from Garlic Using a Combination of Shoot Meristem Culture, Thermotherapy, and Chemical Treatment

**DOI:** 10.3390/pathogens12010129

**Published:** 2023-01-12

**Authors:** Ashwini Prashant Benke, Ram Krishna, Kiran Khandagale, Suresh Gawande, Poonam Shelke, Somnath Dukare, Sweta Dhumal, Major Singh, Vijay Mahajan

**Affiliations:** ICAR—Directorate of Onion and Garlic Research, Rajgurunagar 410505, India

**Keywords:** chemotherapy, virus complex, *Allium sativum* L., Onion yellow dwarf virus, Garlic common latent virus, shallot latent virus, Allexivirus, virus eradication

## Abstract

Garlic (*Allium sativum* L.) is a clonally propagated bulbous crop and can be infected by several viruses under field conditions. A virus complex reduces garlic yield and deteriorates the quality of the produce. In the present study, we aimed to eliminate Onion yellow dwarf virus (OYDV), Garlic common latent virus (GCLV), Shallot latent virus (SLV), and Allexiviruses from the infected crop using combination of meristem culture, thermotherapy, and chemotherapy. In this study, seven different treatments, namely shoot meristem culture, thermotherapy direct culture, chemotherapy direct culture, chemotherapy + meristem culture, thermotherapy + meristem culture, thermotherapy + chemotherapy direct culture, and thermotherapy + chemotherapy + meristem culture (TCMC), were used. Multiplex polymerase chain reaction (PCR) was employed to detect virus elimination, which revealed the percentage of virus-free plants was between 65 and 100%, 55 and 100%, and 13 and 100% in the case of GCLV, SLV, and OYDV, respectively. The in vitro regeneration efficiency was between 66.06 and 98.98%. However, the elimination of Allexiviruses could not be achieved. TCMC was the most effective treatment for eliminating GCLV, SLV, and OYDV from garlic, with 66.06% plant regeneration efficiency. The viral titre of the Allexivirus under all the treatments was monitored using real-time PCR, and the lowest viral load was observed in the TCMC treatment. The present study is the first to report the complete removal of GCLV, SLV, and OYDV from Indian red garlic with the application of thermotherapy coupled with chemotherapy and shoot meristem culture.

## 1. Introduction

Garlic (*Allium sativum* L.) is a bulbous medicinal spice crop, ranked second after onion in *Allium* in terms of production due to its culinary, nutraceutical, and medicinal uses, particularly owing to its antibacterial and anti-inflammatory properties [1,2]. Globally, 28.4 million tons of garlic is produced annually; China is ranked first in garlic production, followed by India (2.9 million tons) (FAO, 2020). However, in the last 2 decades, India has recorded an approximately 3-fold increase in garlic production (0.49 to 1.69 million tons) (FAO, 2018), but the increase in productivity was comparatively smaller (4.16 to 5.27 MT/ha). India has a smaller rate of garlic production compared to other garlic-producing countries due to environmental and geographical differences [3]. The primary limitation in the genetic improvement of garlic is sexual sterility [4,5,6].

The existing commercial garlic cultivars do not produce fertile flowers or viable seeds [5]. Thus, clonal propagation is carried out in garlic using cloves and air bulbils. Clonal propagation makes garlic vulnerable to viral infection since most viruses are transmitted through the vegetative plant materials. Viral infection remarkably deteriorates garlic bulb yield and quality [7,8]. Numerous viruses can infect garlic through different modes of transmission. Garlic can be infected by viruses of various genera, including Potyviruses such as Onion yellow dwarf virus (OYDV), Leek yellow stripe virus (LYSV), and Shallot yellow stripe virus; Carlaviruses such as Garlic common latent virus (GCLV) and Shallot latent virus (SLV); Tospovirus (Iris yellow spot virus, IYSV), and Allexiviruses (Garlic virus-A, Garlic virus-B, Garlic virus-C, Garlic virus-D, Garlic virus-E, and Garlic virus-X) [9,10,11,12,13]. Generally, under field conditions, garlic is infected by multiple viruses, known as the virus complex, which severely reduces the crop yield [14,15,16]. Furthermore, due to vegetative propagation, the viral load increases with each generation [16,17]. Therefore, periodic use of virus-free planting material is an important way to maintain a sustainable garlic yield with good profitability.

Ten garlic-infecting viruses, namely GCLV, IYSV, LYSV, OYDV, SLV, GarV-D, GarV-A, GarV-C, GarV-X, and GarV-B, have been reported in India [13,18,19,20,21,22,23,24,25,26]. Virus-free material can be easily exploited for planting material production in India; however, a national variety of virus-free garlic bulbs is unavailable to date. All Indian garlic cultivars are infected by a virus complex [24,25,26], and planting material transportation within the country plays a vital role in spreading viruses.

Meristem culture is routinely used for eliminating plant viruses because of the high genetic stability, growth potential, and cell division rate of meristems [27,28]. The application of meristem culture in combination with thermos- and chemotherapy can increase the virus elimination frequency. The meristem/shoot tip culture and thermotherapy have been effectively employed for eliminating garlic viruses [13,14,29,30,31,32,33]. Alternative methods such as chemotherapy, cryotherapy, and root meristem culture have also been applied to eliminate virus-infected Alliums [34,35,36,37,38,39]. Therefore, in this study, we evaluated the efficacy of existing meristem-tip-culture-based methods to eliminate or minimize the titre of garlic viruses in a popular garlic variety, Bhima Purple.

## 2. Results

### 2.1. Treatments and Plant Regeneration

The explants were subjected to seven treatments, shoot meristem culture (SMC), thermotherapy direct culture (TDC), chemotherapy direct culture (CDC), chemotherapy + meristem culture (CMC), thermotherapy + meristem culture (TMC), thermotherapy + chemotherapy + direct culture (TCDC), and thermotherapy + chemotherapy + meristem culture (TCMC), in the experiment. The regeneration efficiency varied from 92.5% to 100% in control explants and from 66% to 98.9% in the treated explants. The lowest regeneration efficiency (66%) was reported in plants under TCMC, whereas the highest regeneration efficiency (98.9%) was reported in plants under TDC. Thermo- and chemotherapy significantly reduced the regeneration frequency; furthermore, the combination of thermotherapy with chemotherapy potentially reduced the regeneration efficiency percentage (Table 1. The growth characteristics of the plants also differed across different treatments; the slowest growth was observed in plants under TCMC treatment, whereas the highest growth was observed in plants under TDC, followed by those under CDC (Table 1 and Table S1).

### 2.2. Virus Elimination

To assess the virus elimination efficiency of the treatments, we analysed 60 random samples from each treatment. The percentage of virus-infected plants after all the treatments ranged from 0% to 100% (Table 1); however, none of the treatments could completely eradicate Allexiviruses. TCDC treatment was successful in completely eliminating (100%) the GCLV and OYDV and 66% plantlets were freed from SLV with a regeneration efficiency of 96.4 ± 1.6%. Among these treatments, TCMC was the most effective in terms of eradication of garlic viruses. All the plantlets under TCMC were found to be free from GCLV, OYDV, and SLV. However, the regeneration efficiency was reduced (66%), and the growth of the plantlets in the culture medium was slow. Thus, TCDC and TCMC could be used for efficient generation of virus-free garlic planting material to maintain the good yield of garlic.

### 2.3. Absolute Quantification of Allexivirus

**Figure 1:**
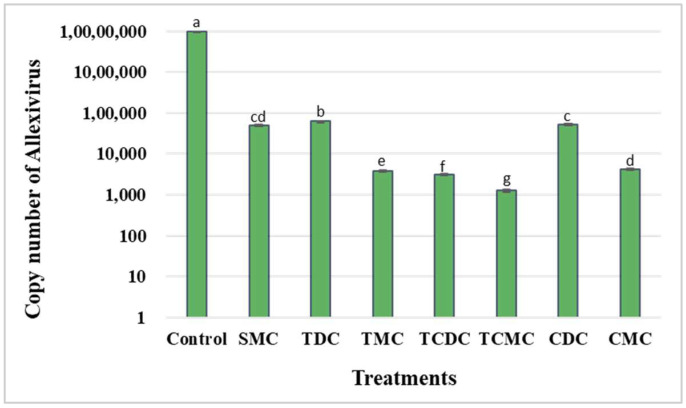
Effect of different treatments on the *Allexivirus* copy number. Means followed by the same letter along same row are not significantly different (*p* ≤ 0.05), according to Duncan’s multiple range test. C: control; SMC: shoot meristem culture; TDC: thermotherapy direct culture; TMC: thermotherapy meristem culture; TCDC: thermotherapy chemotherapy direct culture; TCMC: thermotherapy chemotherapy meristem culture; CDC: chemotherapy direct culture; CMC: chemotherapy meristem culture.

Allexivirus was detected in all treated explants. Hence, we estimated the Allexivirus titre for each treatment through absolute quantification of the copy number using qRT-PCR (Figure 1). TCMC was found to be the most effective treatment, with the lowest copy number (1263.3 ± 60.9), which was 7764.06 times smaller than the copy number in the control plants (9,808,600 ± 89,443). TCDC, TMC, and CMC treatments reduced the copy number of Allexivirus by 3147, 2547, and 2357 times, respectively.

## 3. Discussion

Garlic is a vegetative propagated crop, and thus the accumulation of viruses over generations reduces the quantity and quality of garlic bulbs [14,28,40,41,42,43,44]. In this study, we excluded two major viruses, viz. LYSV and IYSV, because the level of LYSV infection was negligible in our genetic stock, and IYSV is not reported to be transmitted through seed/bulbs [45]. The conventional breeding techniques are not suitable for developing virus-resistant varieties of garlic due to its floral sterility [4,6]. Presently, viruses can be eliminated from these crops through different physical and chemical techniques to obtain virus-free propagating materials [14,27,28,40,41,46]. The efficiency of the technique depends on the type and the accumulation level of a virus in the plant, its physiology, and plants’ resistance level [47]. Techniques such as tissue culture (meristem culture, somatic embryogenesis); cold, chemical, and heat treatments; or tissue culture combined with cold, chemical, and heat treatments have been applied to eliminate viruses [14,28,37,39,48,49,50]. The success of any approach does not depend only on virus elimination but also on the successful recovery of virus-free garlic plants.

Recovery of the treated plants depends on the intensity and duration of the treatments. In the present study, 98.98% and 96.48% of plants were recovered under TDC and CDC, respectively, compared with the other treatments, which may be due to the growth-supporting behaviour of the bulbils under the stem disk dome culture (Table 1). However, the plants under TDC exhibited superior growth to those under CDC, possibly due to the presence of ribavirin in the culture medium in the case of CDC. The findings are congruent with those of a study by Ramírez-Malagón et al. [51] in which the growth of ribavirin-treated (205 µM) garlic was reported to be slow. Similarly, Senula et al. [37] also reported a reduction in regeneration efficiency of garlic in a ribavirin-containing medium. The lowest plant recovery (66.06%) was detected under TCMC (Table 1) because the prolonged heat and ribavirin treatment caused the desiccation of bulbils, which reduced their growth and recovery efficiency; similar findings were reported by earlier studies [39,47,52]. In the current study, growth under TMC and CMC was found to be poor compared with that under SMC. Several techniques with different virus elimination efficiencies have been employed for the production of virus-free garlic. Thermotherapy is the most frequently used technique for virus elimination, as heat slows down the multiplication of different viruses [14,52,53]. Sometimes, thermotherapy is also combined with tissue culture. In the present study, thermotherapy alone exhibited GCLV, SLV, and OYDV elimination efficiencies of 80%, 63%, and 18%, respectively, but failed to eliminate Allexivirus. Pramesh and Baranwal [14] and Conci et al. [53] have reported similar results; the authors treated garlic for 21–40 days at 36 °C, and the treatment resulted in the elimination of 53% of OYDV and LYSV, 67% of GCLV, and 13–18% of Allexivirus. However, contrary to these studies, the present study failed to eliminate Allexiviruses, possibly due to the high initial titre of this virus in the experimental explants (Table 1), as well as the genotype of garlic (Bhima Purple) used in the experiment. The virus titre also varies with the genotype, and the genotype (g = G − 1) used by Pramesh and Baranwal [14] could have had a low titre of the Allexivirus GarV-X, due to which this virus was easily eliminated. They used GarV-X-specific primers for indexing, while here we used Allexivirus-specific primers that could detect all members of Allexivirus. Another possibility is that the technique used for virus screening, RT-PCR, is more accurate and precise than ELISA [54].

In the present study, the elimination of 66% of GCLV, 55% of SLV, and 13% of OYDV was noted when only meristem culture was applied; these findings conform to those of Pramesh and Baranwal [14] and Vieira et al. [39], who reported that the rate of virus elimination was between 20 and 80% depending on the virus type. Meristem culture is frequently combined with thermotherapy to effectively eliminate viruses from vegetatively propagated crops, such as apple (*Malus domestica*), cassava (*Manihot esculenta*), grape (*Vitis vinifera*), garlic, potato (*Solanum tuberosum*), shallot (*Allium cepa gr. Aggregatum*), and sweet potato (*Ipomoea batatas*), and this approach has been shown to be effective in eliminating 20–100% of viruses depending on their type [14,28,40,41,42,43,44,55]. The combination of meristem culture with thermotherapy exhibited a higher elimination efficiency than that seen with the individual treatments. TMC eliminated 85% of GCLV, 85% of SLV, and 20% of OYDV with 91.71% regeneration frequency; however, this treatment failed to eliminate the Allexivirus. Similarly, Pramesh and Baranwal [14] reported that the treatment of garlic at 37 °C, 40 °C, and 42 °C for 7, 14, and 21 days, respectively, eliminated 0–67% of GCLV, SLV, and OYDV, whereas the treatment at 42 °C for 21 days eliminated 33% of GarV-X, with a low (50%) regeneration efficiency and an average regeneration duration of 90 days. Similar findings were reported in a study by Wang et al. [28], in which the elimination of 50–80% of OYDV and SLV was achieved in shallots by coupling thermotherapy and meristem culture.

Antiviral chemicals such as ribavirin and 8-azaguanine exhibited varying plant virus elimination efficiencies depending on the virus type [56,57]. In the current study, ribavirin was used individually and in association with thermotherapy, meristem, and thermotherapy plus meristem treatments for virus elimination. The culture of garlic bulbils in CDC eliminated 65% of GCLV, 55% of SLV, and 35% of OYDV. These findings are consistent with those of Ramírez-Malagón et al. [51], who achieved the elimination of 27–34.8% of garlic potyvirus. Ribavirin eliminates animal and plant viruses and can be effective against both DNA and RNA viruses. Ribavirin inhibits virus replication during the early phase of inoculation [51]. It forms 5′ triphosphate that inhibits the formation of RNA cap [58,59]. The virus elimination efficiency of ribavirin was enhanced in the current study by coupling it with TCDC, CMC, and TCMC treatments (Table 1). The combination of ribavirin with thermotherapy and meristem culture efficiently eliminated viruses by inhibiting virus replication, and preventing virus entry into the meristem from the neighbouring cells. Our results are supported by the findings of Jemal and Feyissa [46], who achieved 100% elimination of Sweet potato feathery mottle virus in sweet potato by using ribavirin and meristem culture. Zhang et al. [43] coupled ribavirin with meristem culture and thermotherapy and successfully eliminated 36–100% of Potato virus Y, Potato virus M, Potato virus X, and Potato virus S from potato. Hu et al. [60] also coupled ribavirin with meristem culture and thermotherapy and eliminated 95% of Apple chlorotic leaf spot virus, Apple stem grooving virus, and Apple stems pitting virus from apple. In the present study, seven treatments were used to eliminate GCLV, SLV, OYDV, and Allexivirus, which potentially reduced the crop yield and quality. TCMC successfully eliminated 100% of GCLV, SLV, and OYDV from garlic and significantly reduced the Allexivirus titre (Figure 1). Allexivirus might reduce garlic yield by 14–32%, depending on its type and complexity [13]. However, the Allexivirus load was significantly reduced (being 7764.06 times smaller than that of the control) in the current study, which is a positive finding for garlic yield improvement. This study is the first to report 100% elimination of GCLV, SLV, and OYDV using a combination of thermotherapy, chemotherapy, and meristem culture techniques, with a high average plant regeneration of 66.06%. However, other techniques, such as root-tip meristem-based methods, must be explored to achieve Allexivirus elimination.

## 4. Conclusions

The study reveals the possibility of achieving elimination of garlic viruses such as GCLV, SLV, and OYDV. The treatments used, thermotherapy, chemotherapy, and SMC, eliminated the viruses at lower rates when applied individually. However, the combination of treatments significantly enhanced the virus elimination rate in infected garlic, and the plant regeneration efficiency was reduced depending on the treatments. In the case of the TCMC treatment, all the garlic plantlets were cured of GCLV, SLV, and OYDV, with 66.06% regeneration efficiency, but the treatment failed to eliminate Allexiviruses. However, TCMC potentially decreased the Allexivirus titre in infected garlic. Achieving the elimination of Allexiviruses is challenging compared to the elimination of Potyviruses and Carlaviruses. Various studies have reported that Allexiviruses affect both the yield and quality of garlic. The present study reports the efficacy of available techniques and protocols for eliminating GCLV, SLV, and OYDV, with commercial values. The elimination of Allexivirus from garlic could not be achieved due to the high titre of these viruses within the garlic host plant. Therefore, a fresh relook into existing methods is needed in view of emerging Allexiviruses infection.

## 5. Material and Methods

### 5.1. Plant Material and Treatments

**Figure 2:**
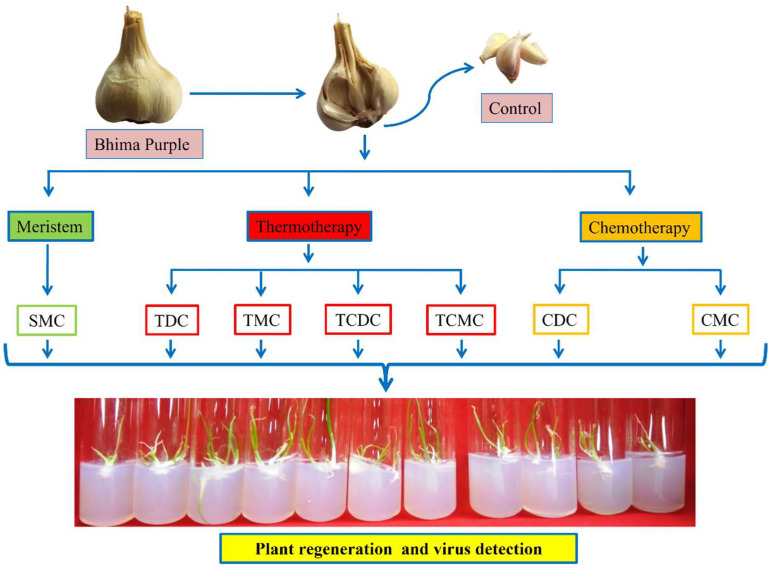
Schematic representation of potyvirus; OYDV, Carla viruses; Garlic common latent virus (GCLV) and SLV and Allexivirus elimination from the Bhima Purple garlic variety. SMC: shoot meristem culture; TDC: thermotherapy direct culture; TMC: thermotherapy meristem culture; TCDC: thermotherapy chemotherapy direct culture; TCMC: thermotherapy chemotherapy meristem culture; CDC: chemotherapy direct culture; CMC: chemotherapy meristem culture.

Bhima Purple, a garlic variety developed at the Indian Council of Agricultural Research-Directorate of Onion and Garlic Research (ICAR-DOGR), was used in the present study. Bhima Purple has purple-coloured bulb skin and is grown in the central garlic-producing states of India. A total of 120 Bhima Purple bulbs, with an average bulb weight of 18–20 g, were selected; 5 cloves separated from each bulb were used for treatment and 2 cloves were used as the control. Each treatment was replicated thrice (for each replicate, 200 cloves for treatment and 80 cloves as control). Thus, a total of 600 cloves were used for each treatment. The following seven treatments were used for virus elimination: (1) shoot meristem culture (SMC), (2) thermotherapy direct culture (TDC), (3) chemotherapy direct culture (CDC), (4) chemotherapy + meristem culture (CMC), (5) thermotherapy + meristem culture (TMC), (6) thermotherapy + chemotherapy direct culture (TCDC), and (7) thermotherapy + chemotherapy + meristem culture (TCMC) (Figure 2).

### 5.2. Media Preparation and Clove Sterilization

MS [61] and B5 [62] (Duchefa Biochemie, Haarlem, The Netherlands) culture media were used. The media were supplemented with 3% (*w*/*v*) sucrose (Duchefa Biochemie, Haarlem, The Netherlands), and the pH was adjusted to 5.8 ± 0.2 by using a pH meter (pH 700, Eutech Instruments, Singapore). Finally, 0.8% (*w*/*v*) agar (Duchefa Biochemie, Haarlem, The Netherlands) was used as the gelling agent, and the media were autoclaved for 15 min at 121 °C. Plant growth regulators were added to the media before or after autoclaving as per the instructions. The cloves were manually peeled and sterilized, as described by Benke et al. [63], with slight modifications. In brief, the cloves were thoroughly washed under running tap water and then treated with 0.1% Bavistin (fungicide, TATA, Mumbai, India) and 0.1% Tween 20 (HiMedia, Mumbai, India) solutions for 15 min with vigorous shaking, followed by rewashing with water. Under the laminar airflow, the cloves were washed twice with sterile distilled water (SDW) followed by a single 70% ethanol wash for 5 min with handshaking. The ethanol was decanted, and the cloves were rewashed with SDW and finally treated with 2% sodium hypochlorite for 15 min. The cloves were washed with SDW thrice and then dried on a sterilized blotting paper.

### 5.3. Methodology of Treatments

In TDC, the garlic bulbs were incubated for 30 days at 37 °C, the upper one-third portion of sterilized cloves was removed, and the remaining portion (consisting of basal disc, first foliar leaf surrounded by storage leaf) was inoculated on the media. In CDC, the sterilized cloves were cut as mentioned above and were cultured on a medium containing 205 μM ribavirin (Duchefa Biochemie, The Netherland), whereas in TCDC, heat-treated sterilized cloves with one-third cut out were cultured on a medium containing ribavirin. In meristem cultures, shoot meristems (0.5–1.0 mm) were isolated by dissecting the sterilized cloves under the Leica stereomicroscope (LEICA MZ6, Leica, Singapore) with sterile forceps and a scalpel and then cultured on the medium. TMC, CMC, and TCMC were achieved by combining the same methodologies used in developing direct culture. The explants in the treatments were inoculated on Petri plates containing MS medium supplemented with naphthalene acetic acid (NAA) (0.1 mg/L) and kinetin (1.0 mg/L). The cultures were incubated at 25 ± 2 °C under 50 μmol m^−2^s^−1^ light intensity, a photoperiod of 16 h, and relative humidity of 60–70%. White fluorescent tube lights (40 W, Philips, Mumbai India) were used as the light source for incubation. After 10 days, 2–4 cm long shoot tips were transferred to B5 medium supplemented with 0.5 mg/L kinetin. The growth of plantlets is categorized into good, average and poor based on plantlet height, bulbil size and weight.

### 5.4. RNA Isolation and Multiplex PCR Reaction

The total RNA was isolated from the 60-day-old treated and control plantlets and used for screening of virus elimination and quantification. According to the manufacturer’s protocol, the total RNA was isolated using the RNeasy plant mini kit (Qiagen, Hilden, Germany) from the 60 random plantlets from each treatment (20 per replicate). The quantity and quality of RNA were analysed using a spectrophotometer (Nanodrop^TM^ND2000, Thermo Fisher, Waltham, MA, USA) and through agarose gel (1%) electrophoresis. Further, cDNA was synthesized from the isolated RNA using the First Strand cDNA Synthesis Kit (Thermo Fisher Scientific, Waltham, MA, USA). The cDNA of each sample was stored at −20 °C until further use.

The multiplex PCR was performed using cDNA as the template and four different virus-specific primers in equimolar concentrations (Table 2). PCR amplifications were carried out in a 15 μL reaction mixture containing 1 μL of template cDNA, 1.5 μL of 10X Taq buffer (Thermo Fisher Scientific, Waltham, MA, USA), 0.35 μL of dNTPs (25 mM) (Thermo Fisher Scientific, Waltham, MA, USA), 0.3 μL of MgCl_2_ (1.5 mM) (Thermo Fisher Scientific, Waltham, MA, USA), 1.2 μL of primers (10 pM) (Thermo Fisher Scientific, Waltham, MA, USA), 0.25 μL Taq-DNA polymerase (Thermo Fisher Scientific, Waltham, MA, USA), and 10.4 μL of sterile Milli-Q water. The PCR was conducted in a ProFlex™ PCR System (Thermo Fisher Scientific, Waltham, MA, USA). The reaction conditions were set as follows: initial denaturation for 5 min; cyclic denaturation of 35 cycles for 30 s at 95 °C; annealing at 55 °C for 1 min, and extension at 72 °C for 1 min (final extension for 10 min). The PCR amplicons were analysed on agarose gel (1.5%) and a 1 kb DNA ladder (Fermentas, Mumbai, India). The amplified products were analysed using a gel documentation system (AlphaImager^TM^ 3400, San Leandro, CA, USA). The amplification was repeated thrice with a multiplex primer to verify the reproducibility of the results.

### 5.5. Real-Time qPCR Reaction

The absolute viral titre of Allexivirus was quantified in all the treatments by using SYBR green-based quantitative real-time PCR with Allexivirus-specific primers [64]. Six samples per treatment were pooled for one qPCR reaction, and the reaction was performed in triplicate. The reaction mixture (20 μL) comprised 10 μL of LightCycler^®^ 480 SYBR Green I Master (Roche, Penzberg, Germany), 0.2 μM primers, and 100 ng of template cDNA. The qPCR was performed in a LightCycler 480 II thermocycler (Roche, Penzberg, Germany). The thermocycler was programmed as follows: initial denaturation at 95 °C for 10 min, followed by 95 °C for 1 min, 40 cycles of cyclic denaturation at 95 °C for 1 min, annealing at 55 °C for 30 s, and extension at 72 °C for 1 min. The standard curve for Allexivirus was generated by a 100-fold serial dilution from 10^9^ to 10^2^ copies (Figure S1). The viral molecule absolute quantification was performed according to the method described by Shafiq et al. [65].

### 5.6. Statistical Analysis

The experiment was set in a randomized complete block design (RCBD), with 3 replications per treatment, and each replicate comprised 280 explants (200 explants for the treatment and 80 explants as the control). The data are represented as mean ± standard deviation. Analysis of variance was performed using SPSS 11.0, and differences in the mean values were compared using Duncan’s multiple range test at a 5% probability level.

## Figures and Tables

**Table 1:** Details of plant regeneration, plant growth behaviour, and percentage of virus-free plants in Bhima Purple garlic variety under different treatments.

Treatments	Code	Plant Regeneration Percent		Plant Growth	Percentage of Virus-Free Plants			
		Control	Treatment		GCLV	SLV	OYDV	Allexi
Shoot meristem culture	SMC	93.0 ± 3.2 ^b^	93.0 ± 2.7 ^b^	Good	66.0 ± 2.2 ^d^	55.0 ± 1.68 ^d^	13.0 ± 0.3 ^e^	0
Thermotherapy direct culture	TDC	100 ± 0 ^a^	98.9 ± 1.0 ^a^	Good	80.0 ± 0.6 ^c^	63.0 ± 2.1 ^c^	18.0 ± 0.5 ^d^	0
Chemotherapy direct culture	CDC	100 ± 0 ^a^	95.0 ± 1.8 ^ab^	Average	65.0 ± 2.1 ^d^	55.0 ± 1.8 ^d^	35.0 ± 0.8 ^c^	0
Chemotherapy + meristem culture	CMC	93.0 ± 1.8 ^b^	87.0 ± 3.6 ^c^	Poor	85.0 ± 0.1 ^b^	65.0 ± 2.3 ^c^	50.0 ± 1.2 ^b^	0
Thermotherapy + meristem culture	TMC	93.0 ± 2.1 ^b^	91.7 ± 3.1 ^b^	Average	85.0 ± 0.2 ^b^	85.0 ± 3.2 ^b^	20.0 ± 0.6 ^d^	0
Thermotherapy + chemotherapy direct culture	TCDC	100 ± 0 ^a^	96.4 ± 1.6 ^a^	Average	100 ± 0 ^a^	66.0 ± 2.2 ^c^	100 ± 0 ^a^	0
Thermotherapy + chemotherapy + meristem culture	TCMC	92.5 ± 3.3 ^b^	66.0 ± 2.0 ^d^	Poor	100 ± 0 ^a^	100 ± 0 ^a^	100 ± 0 ^a^	0

The results are mean ± SE of triplicate measurements. Means followed by the same letter along same column are not significantly different (*p* ≤ 0.05), according to Duncan’s multiple range test.

**Table 2:** Details of primers used for the detection of different viruses in Bhima Purple garlic variety.

Name of Primer	Primer Sequences (5′-3′)	Genome Location	Gene/ Reference	Annealing Temperature	Amplicon Size
SLV-F	AAACCTTTTGGTTCACTTTAGG	7040	JF320811 [14]	57	650
SLV NABP-R	AAYAACATCTAATTCCA	7693		46	
GCLVNABCP-F	AAATGTTAATCGCTAAACGACC	7854	JF320810 [14]	60	586
GCLVNABCP-R	CTTTGTGGATTTTCGGTAAG	8458		56	
OYDV-F	GAAGCACAYATGCAATGAAGG	10,168	NC005029 [14]	59	293
OYDV-R	GCCACAACTAGTGGTACACACCAC	10,451		64	
Allexivirus-F	CYGCTAAGCTATATGCTGAARGG	8623	MN059428 [64]	62	200
Allexivirus-R	TGTTRCAARGTAAGTTTAGYAATATCAACA	8813		60	

Note: F: forward; R: reverse primer, and y: pyrimidine base.

## Data Availability

Data used for the aim of this study have been presented within the text and the Supplementary Materials. Raw data used for statistical analyses are available for further use on request from the corresponding author.

## Supplementary Materials

The following supporting information can be downloaded at: https://www.mdpi.com/article/10.3390/pathogens12010129/s1, Figure S1: Standard curve (A), amplification curve (B), melting curve (C) and melting peaks (D) for the Allexivirus; Table S1: Performance of in vitro plantlets treated for virus elimination, plantlets’ morphological traits, and post-harvest bulbil characterization.
