# Supplementary material for: Efficient Elimination of Viruses from Garlic Using a Combination of Shoot Meristem Culture, Thermotherapy, and Chemical Treatment
Source: Pathogens. 2023 Jan 12;12(1):129. doi: 10.3390/pathogens12010129 (PMC9860850; doi:10.3390/pathogens12010129)
Supplement: Supplementary file 1 [file pathogens-12-00129-s001.zip › Table S1.pdf]

Supplementary Table S1: Performance of *in vitro* plantlets treated for virus elimination, plantlets morphological traits, and post harvest bulbil characterization

**Treatment combination**	**Plant height (cm)**	**Number of roots/plant**	**Number of leaves/plant**	**Leaves overall status**	**Bulbil induction (DAI)**	**Induction of bulbil**	**Bulbil weight (mg)**	**Bulbil equatorial diameter (mm)**	**Bulbil polar diameter (mm)**	**Overall rating on growth parameters**
Shoot meristem culture (SMC)	16.21^{ab}	7^{a}	6^{ab}	Dark green in colour and healthy	72^{ab}	Bulbil induction was late compared to other treatments	617^{ab}	10.48^{a}	10.81^{a}	Good
Thermotherapy direct culture (TDC)	15.85^{ab}	7^{a}	6^{ab}	Dark green in colour and healthy	73^{ab}		532^{abc}	9.72^{ab}	10.55^{a}	Good
Chemotherapy direct culture (CDC)	14.3^{ab}	5^{cd}	6^{cd}	Greenish yellow colour leaf	63^{b}	Bulbil induction observed earlier than SMC & TDC	406^{bcd}	9.09ab	9.40^{ab}	Average
Chemotherapy + meristem culture (CMC)	10.81^{bc}	5^{cd}	6^{cd}	Yellowish leaf and drying observed at the tip	54^{bc}	Bulbil induction was earlier than all other treatments	200^{de}	7.01^{bcd}	7.15^{bcd}	Poor
Thermotherapy + meristem culture (TMC)	14.52^{ab}	4^{bc}	6^{cd}	Greenish yellow colour leaf	62^{b}	Bulbil induction observed earlier than SMC & TDC	406^{bcd}	8.25^{bc}	9.16^{bc}	Average
Thermotherapy + chemotherapy	11.95^{bc}	5^{bc}	6^{bc}	Greenish yellow colour leaf	63^{bc}	Bulbil induction observed	329^{cd}	8.23^{bc}	8.53^{bc}	Average

direct culture (TCDC)						earlier than SMC & TDC				
Thermotherapy + chemotherapy + meristem culture (TCMC)	10.99^{cd}	4^{bc}	5^{bc}	Yellowish leaf and drying observed at the tip	51^{bc}	Bulbil induction was earliest	175^{de}	6.86^{cd}	7.49^{bcd}	Poor

(DAI-Days after Inoculation)
