# Supplementary figures and images for: Efficient Elimination of Viruses from Garlic Using a Combination of Shoot Meristem Culture, Thermotherapy, and Chemical Treatment
Source: Pathogens. 2023 Jan 12;12(1):129. doi: 10.3390/pathogens12010129 (PMC9860850; doi:10.3390/pathogens12010129)

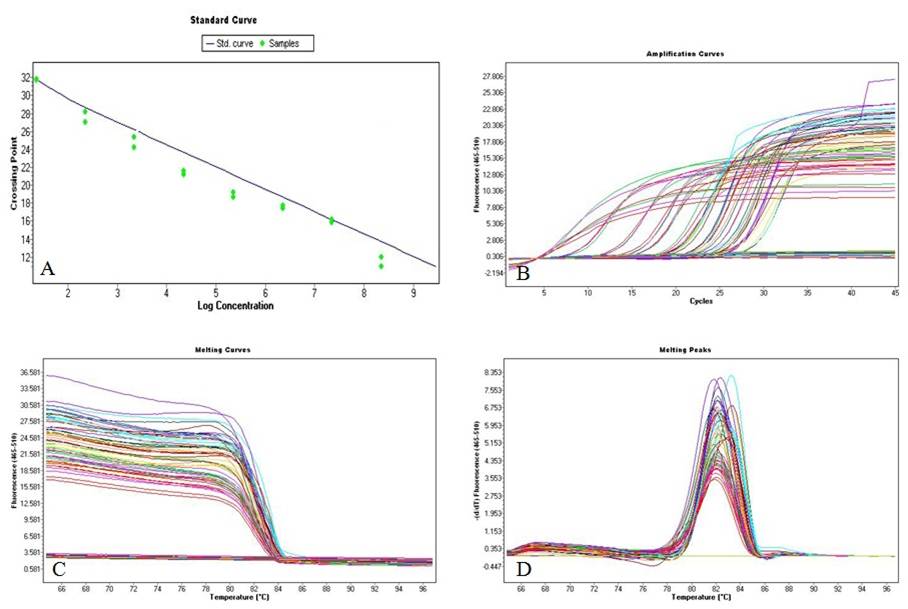

Supplement: Supplementary file 1 [file pathogens-12-00129-s001.zip › supplementary figure 1.JPEG]
